# An Investigation on Micro-Raman Spectra and Wavelet Data Analysis for Pemphigus Vulgaris Follow-up Monitoring

**DOI:** 10.3390/s8063656

**Published:** 2008-06-01

**Authors:** Carlo Camerlingo, Flora Zenone, Giuseppe Perna, Vito Capozzi, Nicola Cirillo, Giovanni Maria Gaeta, Maria Lepore

**Affiliations:** 1 Consiglio Nazionale delle Ricerche, Istituto di Cibernetica “E. Caianiello”, Pozzuoli, Italy; 2 Dipartimento di Scienze Fisiche, Università “Federico II”, Naples, Italy; 3 Dipartimento di Scienze Biomediche, Università di Foggia, Italy; 4 Dipartimento di Malattie Odontostomatologiche, Seconda Università di Napoli, Naples, Italy; 5 Dipartimento di Medicina Sperimentale, Seconda Università di Napoli, Naples, Italy

**Keywords:** blood serum, micro-Raman spectroscopy, wavelet analysis

## Abstract

A wavelet multi-component decomposition algorithm has been used for data analysis of micro-Raman spectra of blood serum samples from patients affected by pemphigus vulgaris at different stages. Pemphigus is a chronic, autoimmune, blistering disease of the skin and mucous membranes with a potentially fatal outcome. Spectra were measured by means of a Raman confocal microspectrometer apparatus using the 632.8 nm line of a He-Ne laser source. A discrete wavelet transform decomposition method has been applied to the recorded Raman spectra in order to overcome problems related to low-level signals and the presence of noise and background components due to light scattering and fluorescence. This numerical data treatment can automatically extract quantitative information from the Raman spectra and makes more reliable the data comparison. Even if an exhaustive investigation has not been done in this work, the feasibility of the follow-up monitoring of pemphigus vulgaris pathology has been clearly proved with useful implications for the clinical applications.

## 1. Introduction

Pemphigus vulgaris (PV) is a group of potentially fatal autoimmune diseases that cause blistering of the skin and oral cavity. It is characterized by disruption of cell-cell adhesion within the suprabasal layers of epithelium, a phenomenon termed acantholysis [1, 2]. Pemphigus vulgaris, the most common type of pemphigus, affects prevalently oral mucosa and skin, but may also involve other mucosae, including nose, conjunctivae, genitals, esophagus, pharynx, and larynx; it is found mainly in middle- aged and elderly individuals. Patients with PV develop IgG autoantibodies against normal constituents of the intercellular substance of keratinocytes, namely desmogleins (Dsg1) and Dsg3. Additional PV autoantigens include acetylcholine receptors (AchRs). The mechanisms by which such autoantibodies induce blisters are not clearly understood. Anti-Dsg3 IgG may impair desmosomal adhesion by exerting direct steric hindrance on Dsg3 extracellular adhesive domains. Alternatively, or additionally, PV IgG may trigger receptor-mediated transduction of intracellular signals finally leading to acantholysis. Pemphigus patients undergo heavy steroideal and immunosuppressant therapy, and follow-up of disease activity may be necessary for a long time (months or years) to monitor the effectiveness of treatment. However, current follow-up measures, including periodic serum drawing and biopsy, are invasive and therefore require a good patients' compliance. In addition, serum titres of circulating autoantibodies, such as anti-intercellular substance (ICS) IgG and anti-Dsgs IgG, do not always mirror the clinical course and actual phenotype of PV [3]. More reliable serum markers of disease activity and progression are therefore needed.

In this paper we investigated the possibility of using micro-Raman spectroscopy for serum analysis for monitoring disease during drug therapy. As well-known Raman spectroscopy on tissue and blood can provide specific information for detecting and diagnosing diseases [4, 5]. Some authors has recently reported that Raman investigation on serum samples can be particularly valuable [6,7]. For this reason we examined samples of blood serum of patients affected by PV, under therapy and in a remission stage of illness. Previous results [8, 9] showed that Raman spectra from pathological and healthy tissues are qualitatively different and the former are dominated by protein contributions while the latter can be attributed mainly to lipid [4, 10]. As far as concerns serum samples their spectra seems to reproduce the main characteristics of tissues spectra, even though they are more affected by noise [9]. For this reason we adopted a wavelet multi-component decomposition algorithm for analysing the Raman spectra of blood serum samples [11]. This data processing method has been already successfully adopted for overcoming problems relative to noise and backgrounds components caused by light diffusion and fluorescence in low-level signals spectra. The linear regression approach that has been used to correlate the Raman spectra informative contents to the different stages of therapy seems to give significant results for serum.

## 2. Materials and methods

### 2.1 Sample preparation and spectra acquisition

Raman spectra were collected on serum samples from patients with histological and immunofluorescence findings compatible with the diagnosis of PV. Patients ( 2 subjects) with detectable titres of anti-ICS IgG and at least two oral/skin lesions were considered as active PV, whereas those taking immunosuppressive agents at the time of biopsy and serum collection were referred to as PV in therapy (2 subjects). Patients (2 subjects) with no clinical evidence for blisters and ICS titres < 1:40 at the end of the therapy were considered as PV in remission (recovered). Informed consent was obtained from all participants. Blood samples were taken by using a butterfly needle and collected into 10 ml blood serum separator tubes under routine conditions. Blood was separated into a lighter phase (serum) and a heavier phase after centrifugation at 1000 × g for 5 min. Serum was transferred to fresh tubes and samples were then stored at -20°C until use. For Raman examination a drop of serum was placed on a microscope glass and covered by a 170 μm thick cover glass, Samples were examined using a micro-Raman spectrometer equipped with a confocal microscope (Horiba-Jobin Yvon). A *He*-*Ne* laser operating at a light wavelength λ = 632.8 nm with a laser maximum delivery of about 3.5mW at sample level was used as exciting source. The spectrograph included monochromator, a CCD detector with a chip size of 1024×256 equipped with a Peltier cell and with a grating of 1800 grooves/mm. The laser light was focused on the sample surface by means of a 50X long working distance optical objective (Olympus MPLAN 50x/0.75) on an excitation spot size with a diameter of about 50 *μ*m. A notch filter (Kaiser Optic) was used in the collimated scattered beam to reduce the laser background. This device was directly controlled by the data acquisition software. A color video camera integrate within the microscope enabled the user to visualize the sample in reflection. For each patient two blood samples were prepared and several spectra were acquired for each samples. The μ-RS spectrum was generally evaluated in the wavenumber shift regions of 1000–1800 cm^-1^ and 2700-3000 cm^-1^ where peaks assigned to vibration modes of amide I (near 1650 cm^-1^), CH_2_ and CH_3_ bending modes (near 1445 cm^-1^, 2850-2930 cm^-1^) and amide III (1240–1260 cm^-1^) are placed [5, 8]. Accumulation times ranging in 60–300 s were used for the μ-RS acquisitions. It should be noted that we have used visible low power laser, in contrast with other authors who prefer near infrared laser source to further minimize fluorescence effects, although the latter approach needs the use of more powerful lasers as reported in literature [5-7, 10].

### 2.2 Wavelet deconvolution

Light dispersion effects due to the presence of liquid in the samples considered, affected the quality of the μ-RS spectra, which featured typically a large background signal and a relatively high level of noise. An automatic numerical data treatment based on wavelet algorithm was used in order to suppress the non-correlated signal, to subtract the background signal and to increase the quantitative readibility of the Raman signal. As reported by Camerlingo et al. [11] this method allows us to extract quantitative information from weak and noising Raman signals as those typically featured by biological samples. The Raman spectrum can be assimilated to a time modulated signal f(*t*) on a finite time interval, where the wavenumber shift stands as the variable *t*. The wavelet algorithm cuts up the signal into different ‘frequency’ components, similarly to the conventional Fourier transform, but it uses spatially localized functions with average zero value (namely wavelets, small waves) instead of conventional sinusoidal functions and makes it possible to have information on both frequency and time dependences. Basically, the signal f (*t*) is represented in terms of the sum of elementary wavelets and decomposed into two signals, one containing the low frequency components (approximation *A1*) and the other the fluctuations (detail *D1*). The algorithm is iteratively applied to the ‘ approximated’ part of the function and a higher level of the *A2* and *D2* component pair is generated. A hierarchical representation of the data set is thus obtained allowing a multi-resolution analysis, known as Discrete Wavelet transform (DWT), in which details or fluctuations of different levels of resolution are represented by the superposition of wavelets with suitable dilation. Starting from the decomposed parts, the signal can be reconstructed by an inverted process known as Inverted Discrete Wavelet Transform (IDWT). If the last approximation component is not included in the IDWT process, the smoother part of the signal will be removed. In the case of a Raman spectrum, this background signal component is mainly caused by light diffusion and fluorescent processes. Similarly, by removing the fast frequency components, namely the low index coefficient set, it is possible to eliminate non-correlated noise signals. In this work biorthogonal wavelets based on the β-spline function were employed [12, 13], using DWT and IDWT program routines of MATLAB 6.5 (by MathWorks Inc.). The decomposition of the signal was performed up to the level n = 8 using biorthogonal wavelets Bior 6.8, with 6th and 8th order filter algorithms involving 17 and 11 data points, respectively. The DWT was applied to the signal which was decomposed into nine data sets (*D1*, …, *D8*, *A8*). The signal was reconstructed by employing only detailed components *D*5– *D*8, causing both excess noise and background signals to be rejected. The validity of the proposed data analysis procedure has been tested on well-characterized Raman spectra of acetylsalicylic acid samples as reported in Ref. 11, where the feasibility of quantitative analysis is also discussed. It can be noted that this procedure does not require any other pre-processing of data and is an automatic numerical algorithm suitable for integration in a friendly interface equipment for users with limited mathematical knowledge.

### 2.3 Linear regression analysis

Linear regression of data is performed in order to compare quantitatively micro-Raman spectra. The spectra here considered are composed by about 800 points which are compared with those of the reference spectrum of sample from recovered patient (end of therapy). A linear fit of the data is performed by using ordinary least squares method and for each spectrum the *R*^2^ correlation coefficient is calculated as the ratio of the sum of squares of differences of reference data with the average value and the sum of squares of differences of observed data with the average value. The *R*^2^ coefficient gives a statistical measure of how well the regression line approximates the real data points and it ranges from 0 for uncorrelated data, to 1 for perfect fit.

## 3. Results and discussion

### 3.1 Wavelet analysis

**Figure 1.**
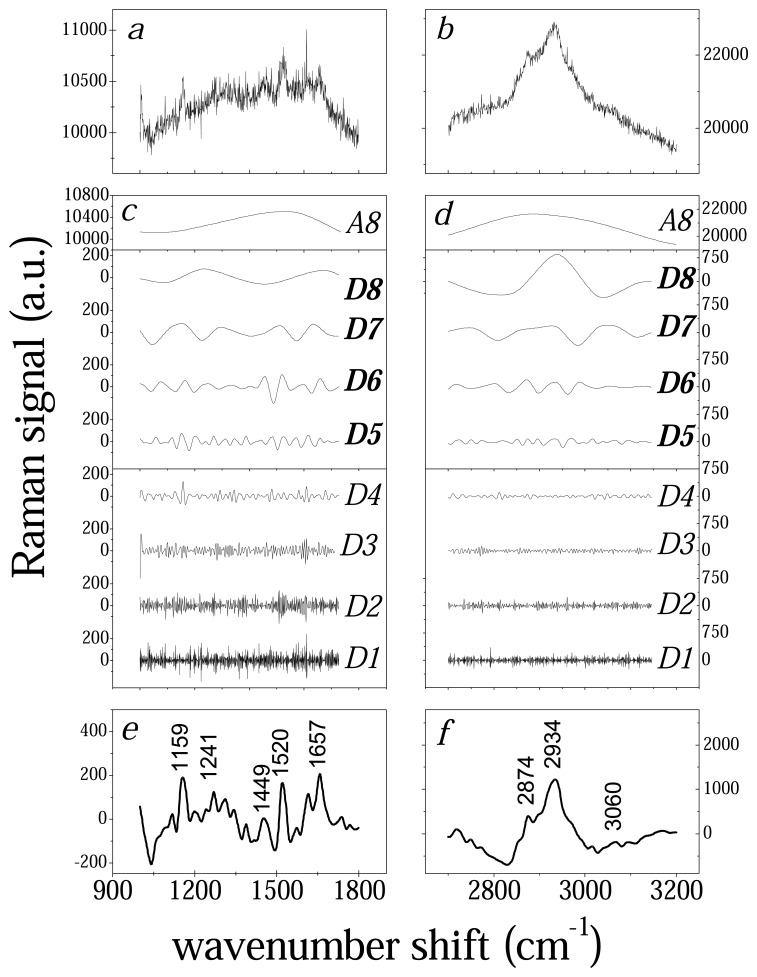
Outline of Raman signal elaboration by wavelet algorithm. The raw data spectra (*a* and *b*) are decomposed by Discrete Wavelet Transform (DWT) in the components *D1*,…,*D8, A8* (*c* and *d*). Using the Inverted Discrete Wavelet Transform (IDWT) the signal is reconstructed from *D5*,.., *D8* components (*e* and *f*).

All data considered in this work have been cleaned numerically from noise and background signal using the wavelet algorithm described in section *2.2*. In Fig.1 is schematically outlined the data treatment performed using as example the data from Raman spectrum collected from a recovered PV patient. The raw data concerning Raman response in the wavenumber shift range of 900-1800 cm^-1^ and 2700-3000 cm^-1^ respectively are reported in Fig. 1 a and 1b. The DWT process is applied and the original signal is decomposed in the curves *D1*,…, *D8*, *A8* (Fig.1 c and d). Finally the spectra reported in Fig.1 e and 1 f are obtained by applying IDWT on data *D5*,*D6*,*D7*,*D8*. The background signal is removed and the major Raman peaks are emphasized. The noise level of signal is significantly reduced with respect to the raw spectrum. In Fig.1 e we see the main structures around 1150-1160 cm^-1^ (C-C stretch and COH deformation), 1241 cm^-1^ (amide III), 1430 –1450 cm^-1^ (CH_3_ deformation), 1520 cm^-1^ and 1657 cm^-1^ (amide I). It is interesting to note that the serum spectra clearly resemble the immunoglobuline spectrum [14] with particular reference to 1159 cm^-1^ and 1520 cm^-1^ peaks that will be further analyzed in the following. The wavelet data treatment is equally efficient on the high wavenumber region of Raman spectra of blood serum samples (fig. 1 f). In this case we are able to evidence the contribution of CH stretch at 2874 cm^-1^, CH_3_ asymmetric stretch at 2934 cm^-1^ and CH olefinic stretch at 3060 cm^-1^.

**Figure 2.**
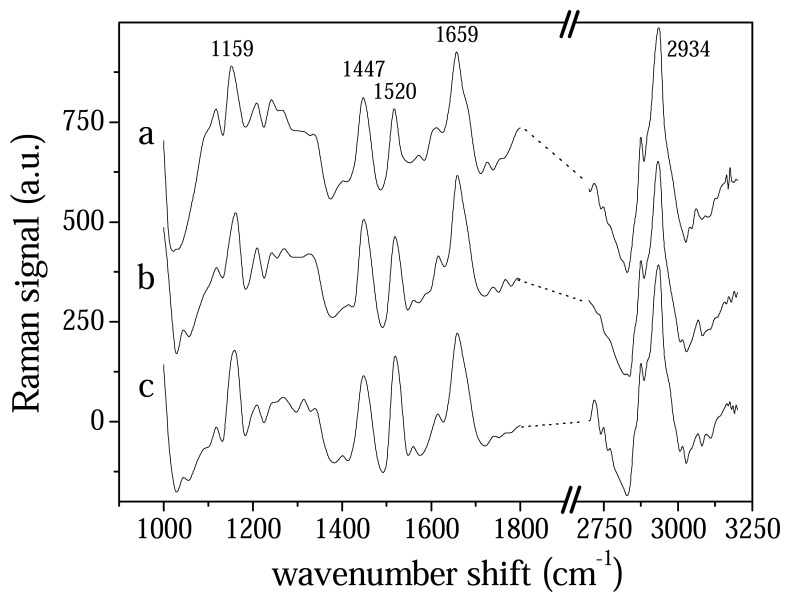
Typical Raman spectrum of blood serum from patients with active PV (a), under drug therapy (b) and from a recovered patient (c).

In fig.2 we reported the spectra of serum samples from patients at different stages of illness after wavelet data treatment. As is evident from fig. 2 the different stages of illness do not cause the appearance and the disappearance of peaks and bands in the Raman spectra but rather the modification of these structures confirming a behavior similar to that of tissue and already reported in literature by Malini et al [10]. These authors compared spectra from normal, malignant, inflammatory and premalignant oral tissue, and noticed that effects of pathologies regard the peak shapes of spectra and the relative intensity among them, more than their position. The complexity of these changes demand to consider the whole spectrum more than single structure for a significant data analysis procedure.

For this purpose a linear regression analysis has been performed. In general the spectrum data sets scale linearly. The relative error found in the determination of the coefficient of proportionality is typically of a few percent, within the regression confidence interval of 95%.

**Figure 3.**
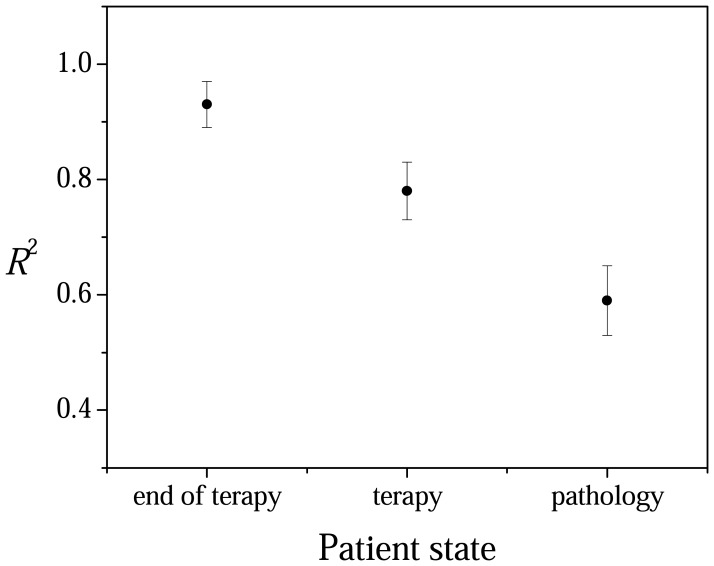
Behavior of *R*^2^ resulting from linear regression of Raman spectra relative to blood serum from patients in the remission stage of illness (recovered), from patients under drug therapy and from PV active patients in the wavenumber 1000-1800 cm^-1^. Dots and bar indicate the mean of *R*^2^ and the error in its determination.

This allows us to exploit in global way the changes that we noticed in the DWT-IDWT processed spectra. In fact when correlation factor is estimated between the spectra related to samples from patients at different stages of illness a decreasing behavior is obtained for the coefficient of correlation, as shown in Fig.3. The spectra acquired from the same kind of samples have typically a relative *R*^2^ coefficient dispersion larger than 0.9.

**Figure 4.**
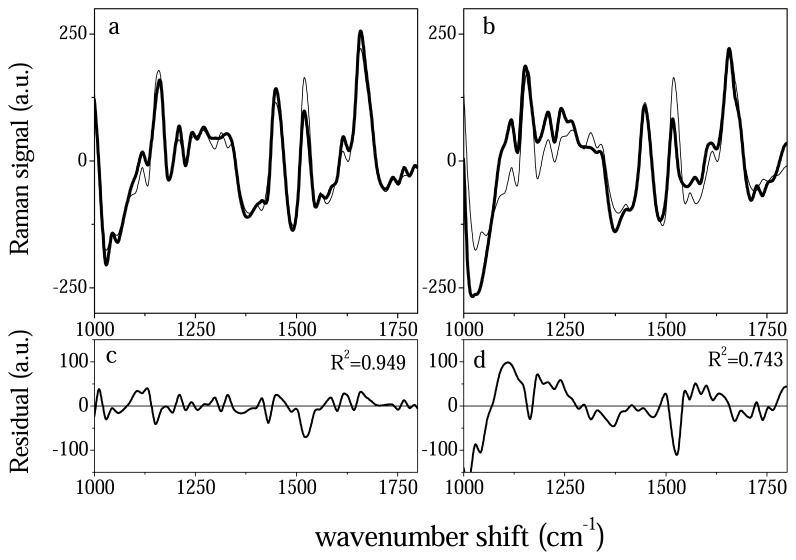
Typical Raman spectrum from under drug therapy (a) and PV active patients (b). The spectra are compared to the spectrum of recovered patient (thin lines) by means of linear regression analysis. The residual signal between signal in (a), signal in (b) and reference signal (from recovered patient) is reported in (c) and (d) respectively.

A deeper insight in follow-up monitoring can be obtained when the contribution of peaks around 1159 and 1520 cm^-1^ is considered. These two peaks that are present in the immunoglobuline spectrum are related to carotene content [15] and seem to be considered the main responsible of differences in the serum samples here investigated. In fact, when comparison is performed between spectra from recovered and active PV patients (see fig. 4a) or from patients under therapy and recovered (see fig. 4b) structures around the above-mentioned wavenumber positions are present in the residual coming from the signal comparison (fig. 4c and 4d). In particular, carotene content is lower in serum from active PV patients, increases in serum from patients under therapy and is maximum in serum from recovered investigation.

## 4. Conclusions

Raman spectroscopy of biological samples (especially liquid ones) is strongly affected by light scattering and fluorescence effects. Wavelet data processing has confirmed to be very useful to overcome some of these problems making possible to obtain useful information even in liquid samples that are usually the more critical ones. In the present case DWT- IDWT has allowed us to obtain spectra with improved signal-to-noise ratio from blood serum samples in different pathological conditions even when the starting spectra were extremely noisy and poorly structured. Consequently a simple but effective linear regression analysis has been successfully performed indicating the possibility of using Raman spectroscopy to get useful information on blood serum. Moreover, varying levels of carotene have been evidenced in samples related to different stages of illness even though more investigations are required to unambiguously relate these differences to pemphigus vulgaris pathology.
